# Unintended pregnancies: a comprehensive cost–benefit analysis of family planning investment in Pakistan

**DOI:** 10.3389/fpubh.2025.1563721

**Published:** 2025-05-19

**Authors:** Aisha Irum, Olan Naz, Muhammad Ibrahim, Adnan Ahmad Khan

**Affiliations:** Research and Development Solutions (RADS), Islamabad, Pakistan

**Keywords:** family planning, cost–benefit analysis, education, immunization, WASH (water sanitation and hygiene), investments–mathematical models

## Abstract

**Introduction:**

Unintended pregnancies, which account for 19.4 to 38.2% of all births, present a significant and pressing public health challenge in Pakistan. Beyond the adverse effects of women’s agency and choice, unsafe abortions, delayed prenatal care, and poor maternal health, they impose substantial economic costs on essential services such as education, healthcare, water, sanitation, and housing infrastructure. We quantified the economic cost of unintended pregnancies and the benefits of investing in family planning. Our findings directly challenge the rationale behind population-based revenue distribution formulas and provide compelling economic evidence for increased family planning budget allocations at both national and provincial levels.

**Methodology:**

This secondary data analysis employs a rigorous methodology, triangulating data from various national and international sources to conduct a comprehensive cost–benefit analysis. Using trend analysis with linear extrapolation, the study projects the economic impact of unintended pregnancies from 2018 to 2035 across key domains, such as schooling, immunization, safe motherhood, and access to safe water and sanitation. Key variables include contraceptive prevalence rate, unmet need for family planning, general fertility rate, under-five mortality rates, social costs per child, school enrollment rates, and per-user cost of family planning.

**Results:**

Our analysis reveals that the additional programming costs in health, education, and water provision from unintended pregnancies outweigh the investment required for family planning interventions aimed at averting them. Specifically, for every dollar invested in family planning, there is an estimated return of approximately USD $23 in combined cost savings. Education costs represent the largest proportion (51%) of these savings, followed by safe motherhood costs (36%), immunization costs (11%), and water/sanitation costs (2%).

**Conclusion:**

We show substantial cost savings from investing in family planning. Some provincial governments have argued that since national revenue distribution is population-based, they would lose funds if they instituted FP programs. We show that these losses are in a few percentage points while their cost savings in social programs would be in hundreds of percentage points while achieving healthier and flourishing populations. Cost–benefit analysis is a powerful tool for policymakers and may be institutionalized in health, population, planning, and finance ministries.

## 1 Introduction

Unintended pregnancies, characterized by pregnancies that are unwanted or mistimed, represent a profound global public health concern. Nearly half (111 million or 48%) of all pregnancies annually in low- and middle-income countries (LMICs) are unintended (1). These include 34% of all pregnancies in Sub-Saharan Africa and 19% in South Asia that are unintended (2, 3). In Pakistan, 19 to 38% of pregnancies are unintended, with the highest proportions being in Balochistan and Sindh (4). In 2012, the unintended pregnancy rate was 93 per 10,000 women aged 15–49 years, resulting in 4.2 million unintended pregnancies, of which 54% were estimated to have ended in induced abortions and 34% in unplanned births (5).

Unintended pregnancies impose further healthcare burdens and economic costs on individuals through various adverse pregnancy outcomes. These lead to neonatal-maternal morbidity or mortality through delayed prenatal care, the risk of abortions and emergency delivery, and missed or suboptimal postnatal care (6, 7). For individuals, they cause additional mental health issues. For societies, they impose macroeconomic costs by necessitating additional investments in education infrastructure, healthcare such as immunization programs, and the need for more water, sanitation, and hygiene (WASH) facilities to accommodate the growing population. However, data on the economic burden of unintended pregnancies is sparse. Where available, these costs include R$4.1 billion (USD $1.85 billion) annually in Brazil, Norwegian Kroner 164 million in Norway, and €293 million in Spain (8–10). In 2006, 64% of such births in the United States were covered by public programs and added USD $11.1 billion to healthcare costs (11).

However, improved access to family planning services can effectively reduce these pregnancies and their associated health and economic costs, resulting in substantial savings for individuals and nations. For example, in Thailand, Ethiopia, and Malawi, each USD spent on family planning saves USD $2.20 in pregnancy-related healthcare costs (12, 13). For Pakistan, for every USD $1 invested in contraceptive commodities and services between 2019 and 2025, the government would save an average of USD $2.3 for Balochistan, USD $3 for Khyber Pakhtunkhwa, USD $5 for Punjab, and USD $3.2 for Sindh in direct healthcare costs (14–17).

Although the United Nation’s 2030 Agenda for Sustainable Development stresses reducing the unmet need for family planning to address unintended pregnancies and their associated costs, local politics may run contrary. In Pakistan, national policy exemplified by the National Finance Commission (NFC) Award, which assigns 82% weight to population when allocating federal resources to provinces, is often cited as a disincentive to funding family planning programs by the provinces.

While earlier studies have broadly quantified the healthcare cost savings of family planning in Pakistan (14–17), this study makes a novel contribution by incorporating a broader cross-sectoral cost–benefit analysis. Given the high proportion of unintended pregnancies in Pakistan and their associated costs, we hypothesize that increased investment in family planning services in Pakistan will significantly reduce unintended pregnancies, improve maternal and child health outcomes, and yield substantial economic benefits. To test this hypothesis, our study addresses the following research question: What are the economic costs of unintended pregnancies in Pakistan, and how do increased investments in family planning impact healthcare savings, education expenditures, and broader social sectors? To answer this, we quantified and projected the economic costs of unintended pregnancies, including those related to education, immunization, safe water and sanitation, and maternal care, and evaluated the potential healthcare savings resulting from increased investment in family planning through a detailed cost–benefit analysis of family planning investments in Pakistan.

## 2 Methodology

The methodology of this study integrates a cost–benefit analysis framework to evaluate the impact of family planning investments in Pakistan by quantifying and projecting the number of unintended pregnancies averted through effective family planning programs and translating these into economic savings across healthcare, education, and infrastructure.

**Figure 1:**
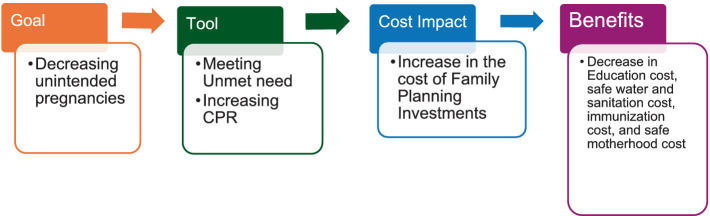
Conceptual framework of cost benefit analysis of family planning.

To guide our cost–benefit analysis of family planning investments, we developed a conceptual framework (Figure 1) that integrates demographic, economic, and health-related variables. The contraceptive prevalence rate (CPR) and unmet need for contraception are central, as they directly influence unintended pregnancy rates, which in turn affect downstream outcomes such as maternal and child health expenditures, education system demands, and economic productivity. Unintended pregnancies lead to increased health sector burdens—rising costs related to maternal care, immunization, and child health services. In the economic domain, these pregnancies reduce female labor participation and increase public welfare spending, while in education, they heighten demand for school infrastructure and support services. Other variables such as population growth rate, GDP per capita, and public health spending help model the broader fiscal and developmental context.

### 2.1 Variables and data

Data for this study were collected from several sources, including the Pakistan Demographic and Health Survey (PDHS 2017–18), the World Bank, the United Nations Population Fund (UNFPA), and peer-reviewed journals. We also incorporated demographic data, healthcare costs, and economic indicators relevant to Pakistan (Table 1). The projections span from 2018 to 2035, with 2018 serving as the base year because the latest available data on contraceptive prevalence rate (CPR) and unmet need for contraception is from PDHS 2018, which are the key variables used to calculate unintended pregnancies.

**Table 1:** Variables and data sources.

Variables	Definition	Assumptions	Sources
Current CPR (married women, any method)	% age of women of childbearing age who use a form of contraception	34%	PDHS, 2018
MWRA	% age of women aged 15–49 who are married	16%	Pakistan Bureau of Statistics, Government of Pakistan
Unmet need (%)	% age of women who want to delay or stop childbearing but are not using contraception	17%	Pakistan Demographic and Health Survey (2006–07,2012–13, 2017–18)
Women who get pregnant (% age)	Percentage of women aged 15–49 who were pregnant at any time during the 12 months preceding the survey	13.8%	PDHS, 2018
Method mix	Percentage share of different contraceptive methods used by MWRA at a given time	Given in Table 3	PDHS, 2018
Failure rate of FP methods	Probability that a woman will become pregnant while using a contraceptive method for one year, assuming she is sexually active and not using any additional contraception	Given in Table 3	Websites: Centers of Disease Control and Prevention Guttmacher Institute
General fertility rate (%)	Number of births per 1,000 women aged 15–49 years in a given year	12	PDHS 2018
Under-5 mortality rate	The probability that a child born in a specific year or period will die before reaching the age of five, if subject to the age-specific mortality rates of that period.	69.4	UNICEF 2018
Abortion rate (%)	Number of induced abortions per 1,000 women of reproductive age (usually 15–49 years) in a given year	2	PDHS, 2018
Discount rate (%)	Rate at which future costs and benefits are adjusted to reflect their present value	23	Stata Bank of Pakistan, 2023
Number of children enrolled (million)		55	Pakistan Education Statistics Report 2021–22 by Pakistan Institute of Education
Net enrollment rate, primary (%)	Percentage of children of official school age for a given level of education who are enrolled in that level	60%	PSLM 2020
Government Education spending per student (% of GDP) (primary)		8.10	World Development Indicators, 2015
Per capita GDP ($US)		1,677	Website of Finance Division, Government of Pakistan, 2021
Cost per fully immunized child (USD)	Total expenditure required to provide all recommended vaccines to a child by age one, including vaccine procurement, delivery, cold chain, and health worker costs	42	Houdroge et al. (21)
Cost per person with safe water and sanitation (USD)	Annual cost per person for simple water supply (standpost) and sanitation (Household sewer connection plus partial treatment of sewage (hardware and software) improvement	16.9	Hutton and Haller (22)
Lifetime Public Education spending per student (USD)		261.9	Authors’ calculations
Cost of FP per user (USD)		19.4	Authors’ calculations
Motherhood cost per child (USD)	Economic, health, and social costs associated with pregnancy, childbirth, and postpartum care for each child a woman bears	106.6	World Health Organization (1999)

The contraceptive prevalence rate (CPR) serves as a key indicator of family planning adoption and its effectiveness in preventing unintended pregnancies. However, despite its importance, a significant unmet need for contraception persists, indicating gaps in access and utilization that limit the full impact of family planning programs. The study also considers the general fertility rate (GFR) and the percentage of women who become pregnant annually, which help estimate the potential impact of increased contraceptive use on reducing unintended pregnancies.

### 2.2 Cost–benefit analysis framework

For the cost and benefit analysis of family planning investments in Pakistan, we adapted the Impact 2 methodology developed by Marie Stopes International (18). This approach estimates the cost of family planning required to avert unintended pregnancies and compares it with the social costs that would arise if these pregnancies resulted in live births. This tool measures the number of unintended pregnancies averted (19) and translates these into cost savings in healthcare, education, and safe water and sanitation. The analysis is grounded in the proximate determinants of fertility, as outlined by Bongaarts (20), which include contraceptive use, unmet need for contraception, and fertility behavior. These determinants directly influence reproductive outcomes and are central to the functioning of the Impact 2 model. The tool relies heavily on contraceptive prevalence rate (CPR), effectiveness of contraceptive methods, and age-specific fertility and marital status data—all of which are key proximate variables affecting fertility and unintended pregnancy rates. Using this framework, we based our projections on the most recent nationally representative data available—PDHS 2017–18. The cost savings are projected through 2035 using linear extrapolation and trend analysis, adjusted for inflation and discounted at a 23% discount rate. This approach ensures that future savings are accurately represented at present, accounting for both the time value of money and inflation. A detailed description of the methodology used to calculate the present value of these savings is given below.

*Step I: calculating the number of unintended pregnancies*.

The first step involves estimating and projecting the number of unintended pregnancies resulting in live births that could have been averted through an effective FP program. This is calculated as follows:

*Unintended Pregnancies and Live Birth = MWRA*^1^ *with Unmet Need* ^2^* (1- % Infant Mortality Rate) * [1- (Abortion Rate+ Miscarriage rate+ rate of still births)].

MWRA with Unmet Need = MWRA * % Unmet Need* General Fertility Rate.

Using the unmet need of 17% and a general fertility rate^3^ of 13.8% for the year 2018, the number of MWRA with unmet needs was calculated. The unmet need was projected to decline by 0.5% annually, reaching 8.5% by 2035. This projection was is based on an observed 8% decrease over 11 years (2007–2018), averaging a 0.7% annual decline. By adopting a more conservative estimate of 0.5%, the projection accounts for potential implementation challenges, making it both realistic and achievable.

*Step II: estimating per child social costs*.

The second step in the analysis involves calculating the social costs that could be avoided if unintended pregnancies were prevented through effective family planning programs. All these costs have been adjusted for inflation^4^ and are presented in U.S. dollars. These costs encompass a wide range of economic and societal impacts; however, we analyze the following areas:

a. *Lifetime per child cost of education per year*: this cost represents the annual financial public investment required to provide education to each child enrolled from early childhood (primary level) to the completion of their education (postgraduate).

*Education Cost = Total Number of Children Enrolled*^5^ * Inflation Adjusted Lifetime Education Cost per Child.

Where,

Total Number of Children enrolled = Children alive at 5 years of age * Primary Enrollment Rate.

Children Alive at 5 years of age = Unintended Pregnancies and Live Birth * (1- % Under 5 Mortality Rate).

Net enrollment rates for each level of education were used to calculate lifetime education costs per child. The total number of students includes those from both public and private institutions. Using per-child lifetime education cost adjusted for inflation, the total discounted lifetime cost of education for unintended live births has been calculated (Table 2).

b. *Cost of immunization per fully immunized child*: expenses associated with ensuring that a child receives all necessary vaccinations to protect against preventable diseases, contributing to overall public health and reducing future healthcare costs. Immunization cost per child is approximately USD $42, as calculated by tracking expenses from vaccine importation to delivery, categorized into salaries, transport, cold chain logistics, and other system costs, weighted by delivery method and location (21). This cost is used to calculate the inflation-adjusted total discounted cost of immunization.

**Table 2:** Important indicators to estimate cost of education.

Net enrollment rate (2020)	
Primary	60%
Middle	21%
High	13%
Higher secondary	4.2%
Undergraduate	1.3%
Number of students (2020–21)	
Public	29,359,376
Private	25,511,588
Education expenditure (in million Rs.)	
Total education expenditure	802,226
Education expenditure (primary)	255,277

Immunization Cost = Unintended Pregnancies and Live Birth * Inflation Adjusted Lifetime Costs of Immunization per Child per Year

c. *Cost per person of access to safe water and sanitation*: providing safe water and adequate sanitation facilities is crucial for maintaining public health and preventing disease. We have used annual cost per person (USD $17) for simple water supply (standpost) and sanitation [Household sewer connection plus partial treatment of sewage (hardware and software)] improvement, calculated by Hutton and Haller (22).

Safe Water and Sanitation Cost = Unintended Pregnancies and Live Birth * Inflation Adjusted Lifetime Costs of Water and Sanitation per Year

d. *Safe motherhood cost per birth*: this cost encompasses all medical and support services required to ensure the health and safety of both mother and child during pregnancy, childbirth, and the postpartum period.

For calculating the motherhood cost of unintended pregnancies, we used the per-child motherhood cost of USD $106.6, calculated by Weissman et al. (23), adjusted for inflation.

Safe Motherhood Cost = Unintended Pregnancies and Live Birth * Inflation Adjusted Lifetime Costs of Motherhood per Child per Year.

*Step III: Calculating per user family planning costs*.

The per-user cost of family planning includes the weighted cost of FP in both public^6^ and private sectors during 2020. The private sector cost of FP per user includes both the social franchising (SF) cost and the social marketing (SM) cost of family planning, calculated using the following formula:


$$\begin{array}{l} \mathrm{FP}\;\text{Cost}\;\mathrm{per}\;\text{user}\;(\mathrm{SF}+\mathrm{SM})=\text{Total spending}\;(\mathrm{SF}+\mathrm{SM})\\ /\text{Total CYPs}\;(\mathrm{SF}+\mathrm{SM}) \end{array}$$

To calculate the per-user cost of social franchising, we used total spending by Marie Stopes International UK. For social marketing, we used total spending on FP by Green Star (24). Data on Couple Years of Protection (CYPs) for various contraceptive methods were taken from the Annual Contraceptive Report 2019–20 by the Pakistan Bureau of Statistics. Finally, these costs were adjusted for inflation using the dollar-adjusted inflation rate and projected through 2035.

a. FP investment required to avert unintended pregnancies

FP cost per user has been used to estimate and project the amount of family planning investments required to avert unintended pregnancies resulting in live births.

Family planning investments = Unintended Pregnancies and Live Births * Inflation Adjusted Lifetime FP Costs per User

b. Savings incurred and lost due to unintended pregnancies

This study comprehensively estimates the savings incurred and the savings lost in terms of births averted using the per-user cost of FP. The focus is on achieving the target Contraceptive Prevalence Rate (CPR) of 50% under the FP 2030 initiative, with projections made both backward and forward.

The key metric, Births Averted, is calculated using the formula:

Births Averted = No. of MWRA * CPR* FP methods Efficacy* General Fertility rate.

Where:

*MWRA* refers to the number of married women of reproductive age.

*FP Methods Efficacy* represents the effectiveness of family planning methods, calculated as:

*FP Methods Efficacy = 1- (Number of MWRA using the method and getting pregnant/Total number of MWRA using the method).*

This efficacy rate is derived using the failure rates of various contraceptive methods, as reported in the Pakistan Demographic and Health Survey (PDHS) (Table 3).

**Table 3:** Important indicators to estimate FP methods efficacy.

FP methods	Methods mix	Failure rate
Condom	27.14%	13%
Pills	5.01%	7%
Injections	7.37%	4%
IUD	6.19%	0.80%
Female sterilization	25.96%	0.50%
Traditional methods	27.14%	14.0%
Implants	1.18%	0.10%

Savings Lost is determined based on the number of unintended pregnancies that could have been averted if the CPR target was achieved. It is calculated as:

Savings Lost = No. of Unintended Pregnancies * per user cost of FP.

Savings Incurred refers to the economic benefits realized by achieving the CPR target and is computed as:

Savings Incurred = No. of MWRA * CPR *per user cost of FP.

The number of MWRA using FP methods is estimated using the Method Mix data from the PDHS.

## 3 Results

### 3.1 Unmet needs and unintended pregnancies

**Figure 2:**
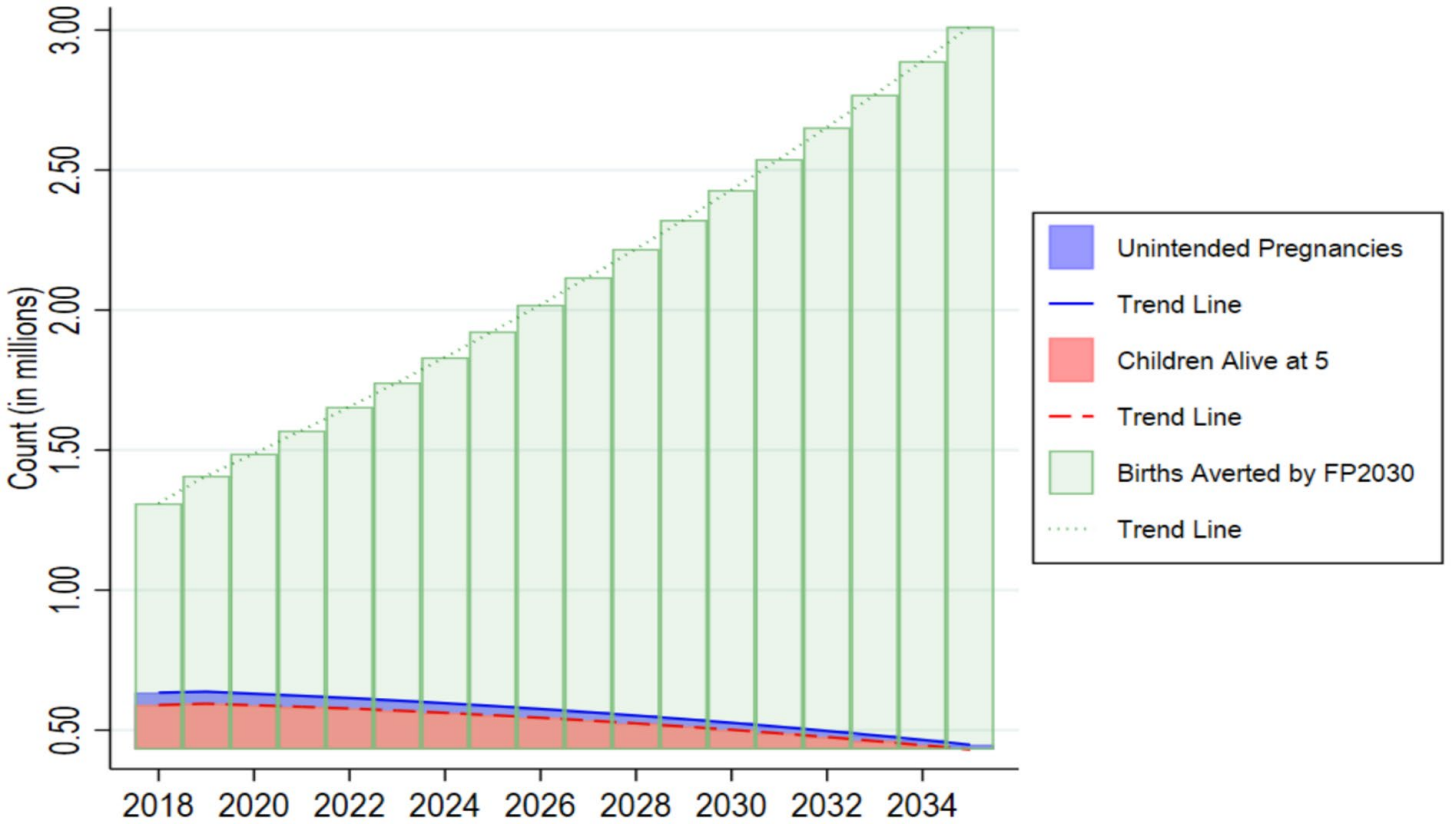
Unintended pregnancies, children alive at 5 and births that could be averted by 2030.

In 2018, the unmet need of 17% led to approximately 0.63 million additional unintended live births annually, of which 0.59 million children would have survived to age five. Our study estimated that if the unmet need for family planning declines every year by 0.5% to reach 8.5% by 2035, it will lead to a 41% reduction in unintended pregnancies from 2018 levels, or 0.4 million additional births a year, or 10.09 million unintended pregnancies cumulatively by 2035 (Figure 2). Among these, the number of children surviving to age five is projected to decrease from 0.6 million to 0.4 million between 2018 and 2035. This implies that the cumulative number of children surviving to age five is expected to reach 9.54 million. These projections are aligned with the decline in the under-five mortality rate, which is projected to decline from 69.4 per 1,000 live births in 2018 to 38.9 by 2035.

### 3.2 Impact of CPR on birth aversions

The CPR of 34% in 2018 helped avert 1.3 million unintended births, while achieving the FP2030 target of 50% CPR by 2030 could avert 2.4 million unintended live births in 2030, increasing to approximately 3 million by 2035 with a projected CPR of 57%. Under the assumed average scenario, where the CPR increases at 0.17% annually, 37.9 million unintended pregnancies would be averted cumulatively for all years between 2018 and 2035.

### 3.3 Projecting social costs of unintended pregnancies

**Figure 3:**
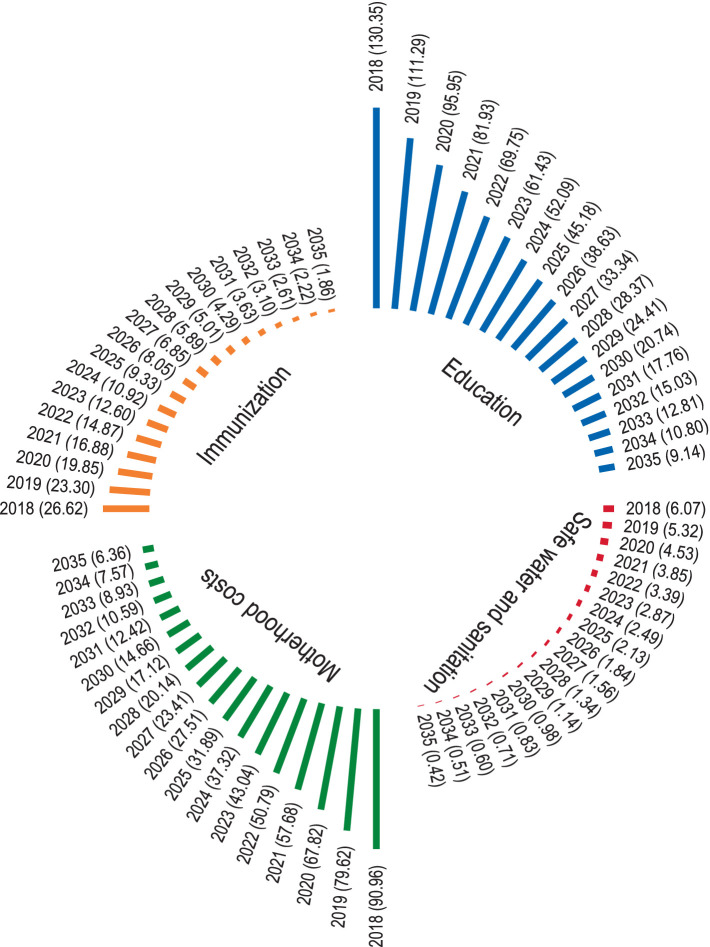
Projected discounted costs of unintended pregnancies.

Our analysis further projects discounted costs associated with unintended pregnancies across education, immunization, safe water and sanitation, and safe motherhood (Figure 3) from 2018 to 2035. Under the current scenario, which includes unintended pregnancies, cumulative education costs account for 51% of the total projected costs, followed by safe motherhood at 36%. In 2020, with a school enrollment rate of 60%, approximately 0.35 million children from unintended pregnancies are estimated to be enrolled in school, incurring an education cost of USD $145 million. This cost is projected to rise to USD $308.6 million by 2035. Immunization costs are also expected to increase from USD $26.6 million in 2018 to USD $62.8 million by 2035. Similarly, access to safe water and sanitation is projected to decline from 0.7 million to 0.5 million people due to population growth from unintended pregnancies, while the associated costs will increase from USD $6 million in 2018 to USD $14.3 million by 2035. Safe motherhood costs are projected to increase from USD $90.9 million in 2018 to USD $214.6 million by 2035. If the unintended pregnancies were averted through improved family planning, substantial savings could be achieved across these domains. A total of USD $1,685.2 million in cumulative discounted costs could be saved from 2018 to 2035, disaggregated as follows: USD $858.9 million in education, USD $177.9 million in immunization, USD $40.6 million in water and sanitation (WASH), and USD $607.8 million in safe motherhood.

### 3.4 Family planning investment and social cost savings

**Figure 4:**
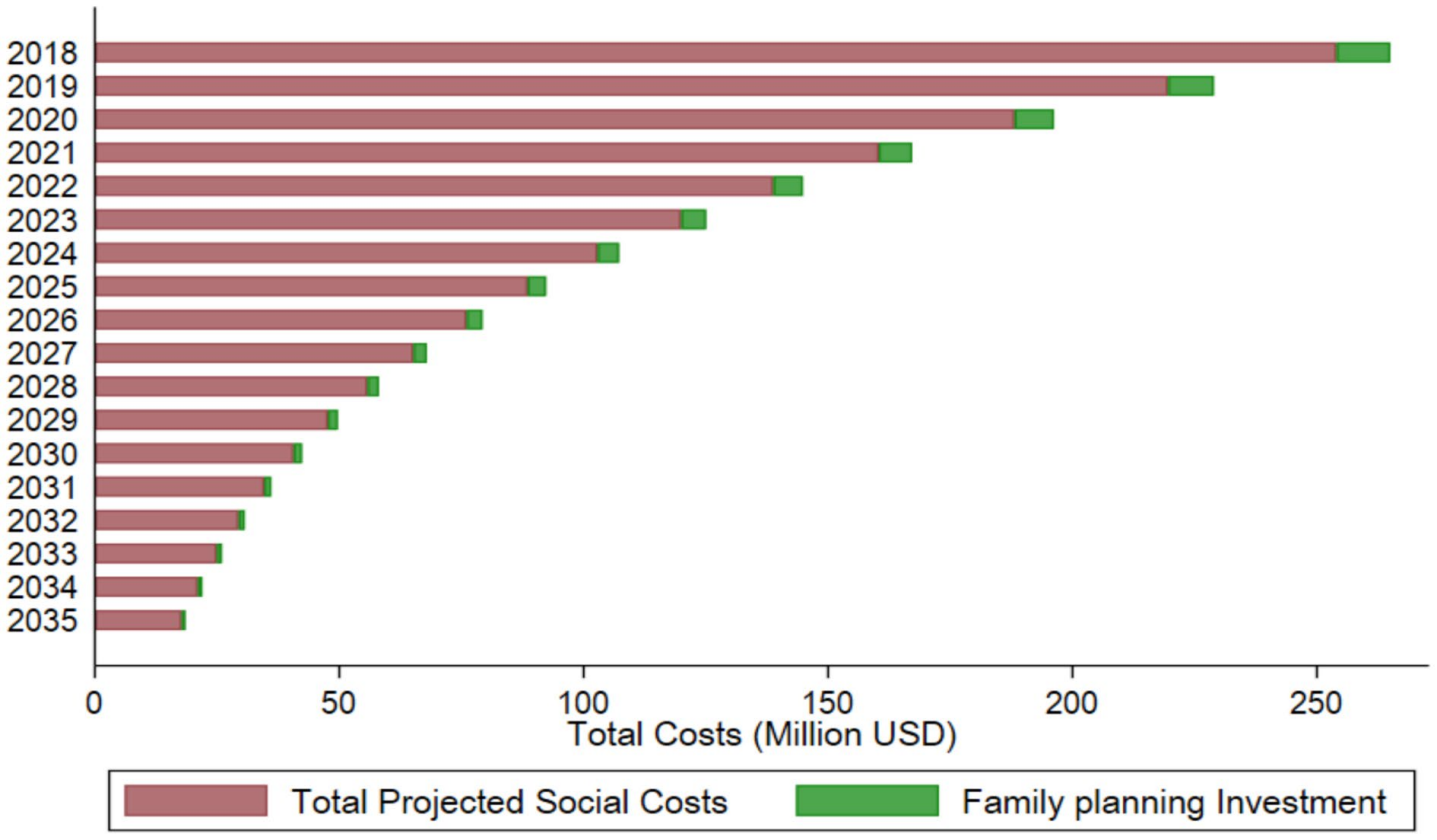
Comparing family planning investment and social costs of unintended pregnancies.

To avert unintended pregnancies, we further calculated the family planning cost per user to be USD $19.42 in 2020 (Table 4). Based on this, the total projected discounted cost of FP required to avert unintended pregnancies is USD $11 million in 2018, declining to USD $0.76 million in 2035. This implies that a cumulative investment of USD $67.08 million between 2018 and 2035 would result in cost savings of USD $1.55 billion across the aforementioned domains by preventing an estimated 9.5 million unintended pregnancies (Figure 3). This implies that every USD $1 allocated to family planning yields approximately USD $23 in savings across education, immunization, safe water and sanitation, and safe pregnancy costs. This is found to be significantly higher than the cost of family planning investments required to avert these pregnancies (Figure 4).

**Table 4:** Important indicators to calculate total weighted FP cost per user (in US dollars).

Sector	No. of FP users (in millions)	Weights	FP cost per user (in USD)	Weighted cost per user (USD)
Public	1.65	33%	28	9.31
Private	2.26	46%	17	7.73
Self-Procurement	1.05	21%	11.2	2.37
Total	4.96	100%		19.42

Source: authors calculations.

### 3.5 Savings lost due to unintended pregnancies

**Figure 5:**
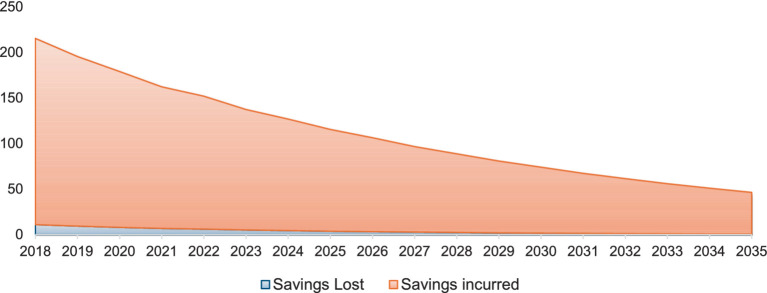
Savings lost and savings gained due to unintended pregnancies (in USD).

We project the financial impact of family planning efforts in Pakistan in terms of savings lost due to unintended pregnancies that could not be averted and savings incurred by successfully preventing such pregnancies. The analysis indicates that if unintended pregnancies are not averted, the projected financial savings lost will accumulate to approximately USD $72 million over the period from 2018 to 2035. Figure 5 highlights the economic burden associated with failing to achieve the target Contraceptive Prevalence Rate (CPR) of 50% under the FP 2030 initiative. Conversely, the study projects significant financial benefits from successfully averting unintended pregnancies through effective family planning. By 2035, the cumulative savings incurred through effective family planning measures will reach USD $182 million.

## 4 Discussion

The study reveals that reducing the unmet need for family planning from 17% in 2018 to 8.5% by 2035 could avert 10.1 million unintended pregnancies, representing 41% fewer unintended pregnancies between 2018 and 2035. This would reduce the added financial burden of these pregnancies of a cumulative USD $1.7 billion across sectors such as education (51% of the costs), maternal healthcare (36%), immunization, and safe water and sanitation. An investment of USD $72.5 million in family planning is needed to avert these unintended pregnancies and would generate net savings of USD $1.5 billion. This implies that every $1 spent on family planning yields an estimated USD $23 in savings across these domains over the next 18 years.

### 4.1 Implications for Pakistan’s education sector

For a country such as Pakistan, where 32% of all children between the ages of 5 to 16 years are out of school, and learning outcomes for school-going children are poor (25, 26), unintended pregnancies place additional strain on an already struggling education system. Since education is a cornerstone of societal development, shaping individuals’ abilities to achieve essential goals, fostering progress (27), and preparing youth for active participation in societal growth and economic productivity (28), these unintended pregnancies limit the economic growth of the nation and lead to despair that contributes to societal discontent and, in some cases, insecurity or extremism (29, 30).

### 4.2 Burden on healthcare system

Similarly, Pakistan’s healthcare system faces challenges that limit its ability to provide adequate and efficient healthcare services to its citizens. One of the significant challenges is insufficient funding. Pakistan spent 1.2% of its gross domestic product (GDP) on the public health sector in 2020–2021 and 1.1% in 2019–2020, despite a population growth rate of 2.6% (31). In per capita terms, it spends approximately USD $ 38 on healthcare, which is much lower than developing countries such as India, the Philippines, and Ghana. Unintended pregnancies impose additional costs on public health systems by increasing the demand for prenatal care, delivery, infant care, immunization, and more. For example, full immunization costs USD $ 42 per child (21). Our results are supported by studies by H, S.-D., M. RA, and L. SR (11) and Monea and Thomas (32), according to which pregnancies cost USD $11–12.6 billion in the US, while in Nepal, the largest burden comes from delivery services (10). Similarly, broader LMIC estimates suggest immunization costs could reach $76 billion over a decade (33).

### 4.3 Water and sanitation challenges

Beyond healthcare, Pakistan is facing significant water stress. The scarcity of clean water, particularly in Sindh and Balochistan, is exacerbated by population growth from unintended pregnancies, putting further strain on existing water and sanitation infrastructure (34–36). The need to expand infrastructure to accommodate population growth diverts resources away from maintaining and improving existing systems, resulting in worsening public health outcomes and increased economic strain (22).

### 4.4 Global and regional comparisons

Globally, publicly funded FP services saved $13.6 billion in the US in 2010, yielding $7.09 in savings per dollar spent (37, 38). Research from LMICs shows that preventing unintended pregnancies—8 million out of 9.5 million in 12 LMICs—via modern contraceptives redirects resources to healthcare, education, and social services (39). Similar investments in sub-Saharan Africa have reduced economic strain on public services (40). Although Pakistan’s FP programs hold even greater potential, the country’s average FP programming costs remain relatively high for both the public and NGO sectors. However, scalable and cost-effective alternatives do exist within Pakistan. For example, the Aapis Initiative, implemented by the Akhter Hameed Khan Foundation in an urban informal settlement in Rawalpindi, demonstrated the effectiveness of a community-driven, demand-and-supply integrated model. Local women were trained as outreach workers (Aapis), delivering household counseling, contraceptives, and referrals, leading to a significant increase in CPR from 33 to 44% and a rise in long-acting reversible contraceptive (LARC) use from 1 to 4% (41). This approach empowered local women, used data for real-time program corrections, and built a sustainable FP ecosystem within underserved communities. These models, operating at a fraction of the cost of conventional programs, illustrate the potential for greater cost-efficiency and impact. A more comprehensive integration of such models into national FP strategies could multiply the returns on investment—potentially increasing savings by three to five times—while promoting local ownership and sustainability. Future studies should explore how such approaches can be scaled nationally to transform Pakistan’s FP outcomes (42).

### 4.5 Broader development benefits

Beyond cost savings, FP promotes sustainable development by enhancing life expectancy, reducing mortality, and improving economic stability (14–17, 43). It also mitigates educational disruptions for young women, breaking cycles of poverty and dependence while contributing to broader socioeconomic progress (44).

## 5 Limitations of the study

One of the primary limitations of this study pertains to the availability of updated data on motherhood costs. Unfortunately, the most recent data on these costs was unavailable, so the study had to rely on estimates provided by Weissman et al. (23). Second, the last DHS was conducted in 2017 and serves as the major source of data for this study. Ideally, we would like to have used a more recent data source and based our projections on that going forward. However, our methodology elucidates such analysis for the future, and the 2018–2035 period of our study can be used as an illustrative example. Finally, as argued above, our analysis is based on relatively high national average costs for family planning. If programs adopt more localized and cost-effective delivery models (42), the potential savings could increase by three to five times.

Considering these limitations, future studies should prioritize the integration of updated FP and maternal health cost data as they become available. Emphasis on real-time monitoring systems and adaptive costing models may also allow for more responsive and accurate forecasting in similar research moving forward.

## 6 Conclusion

Our study highlights the critical need to revise policies that inadvertently encourage population growth by allocating public resources primarily based on population size. Such frameworks not only discourage investments in family planning but also place undue pressure on already overburdened public services, diverting resources away from essential sectors such as health, education, water, and sanitation.

We argue that although some provinces fear a reduction in their share of the National Finance Commission (NFC) award, they continue to underfund family planning programs. However, the marginal gains in budget shares—often only a few percentage points—are vastly outweighed by the enormous costs incurred from unintended pregnancies. Between 2017 and 2035, provinces could save over 2,300% in basic health and WASH (water, sanitation, and hygiene) expenditures by effectively addressing unmet needs for family planning. These savings translate not only into fiscal efficiency but also into broader human development, improved maternal and child health outcomes, and enhanced economic stability.

The evidence presented affirms that family planning is not merely a health intervention—it is a high-return investment in sustainable development. The cost–benefit analysis highlights the transformative potential of such investments, showing that for every USD $1 spent on family planning, up to USD $23 can be saved across critical development sectors. This return is particularly compelling for resource-constrained settings such as Pakistan, where public services are already stretched thin.

To fully leverage these benefits, it is imperative that cost–benefit analyses of family planning and related interventions be institutionalized within key government ministries, including health, population, planning, and finance. One potential mechanism could be the establishment of a National Family Planning Costing Unit within the Ministry of Planning to generate, consolidate, and apply cost-effectiveness evidence in real-time policymaking. Embedding such evidence-based decision-making frameworks can support the development of equitable and efficient policies, helping to align fiscal priorities with national development goals.

Ultimately, prioritizing investments in family planning is not only an economic necessity but also a moral imperative—one that supports population wellbeing, fosters human capital development, and lays the foundation for a more prosperous and resilient Pakistan.

## Data Availability

The original contributions presented in the study are included in the article/supplementary material, further inquiries can be directed to the corresponding author.

## Footnotes

^1^Married Women with Reproductive Age.

^2^According to WHO, “Women with unmet need are those who are fecund and sexually active but are not using any method of contraception, and report not wanting any more children or wanting to delay the next child.”

^3^Percentage of women who get pregnant.

^4^The $ Adjusted inflation rate is calculated by adjusting the inflation rate based on changes in the exchange rate, reflecting the impact of currency value shifts.

^5^The children include those enrolled in both public and private institutions.

^6^The study has used FP cost per user in the public sector calculated in the unpublished work of Research and Development Solutions.

## References

[1] Sully E. A. Biddlecom A. Darroch J. E. Riley T. Ashford L. S. Lince-Deroche N.., Adding it up: investing in sexual and reproductive health 2019, New York, NY: Guttmacher Institute. (2019), 1–56.

[2] Bain LE Zweekhorst MB de Cock Buning T. Prevalence and determinants of unintended pregnancy in sub–saharan Africa: a systematic review. Afr J Rep Health. (2020) 24:187–205. doi: 10.29063/ajrh2020/v24i2.18 34077104

[3] Sarder A Islam SM Maniruzzaman TA Ahammed B. Prevalence of unintended pregnancy and its associated factors: evidence from six south Asian countries. PLoS One. (2021) 16:e0245923. doi: 10.1371/journal.pone.0245923 33524018 PMC7850499

[4] Habib MA Raynes-Greenow C Nausheen S Soofi SB Sajid M Bhutta ZA. Prevalence and determinants of unintended pregnancies amongst women attending antenatal clinics in Pakistan. BMC Preg Childbirth. (2017) 17:1339. doi: 10.1186/s12884-017-1339-z, PMID: 28558671 PMC5450067

[5] Naz L Bari KM Khan JA. Women’s autonomy and unintended pregnancy among reproductive age women in Pakistan. ICF International. (2023).

[6] Ali SA Ali SA. Unmet need for contraception and unintended pregnancies among women of reproductive age group: a situation analysis. Elective Med J. (2014) 2:259–65. doi: 10.18035/emj.v2i3.242

[7] Yazdkhasti M Pourreza A Pirak A Abdi F. Unintended pregnancy and its adverse social and economic consequences on health system: a narrative review article. Iran J Public Health. (2015) 44:12–21. PMID: 26060771 PMC4449999

[8] Henry N Schlueter M Lowin J Lekander I Filonenko A Trussell J. Cost of unintended pregnancy in Norway: a role for long-acting reversible contraception, in journal of family planning and reproductive. Health Care. (2015) 41:109–15. doi: 10.1136/jfprhc-2014-100878, PMID: 25537792 PMC4369438

[9] Lete I Hassan F Chatzitheofilou I Wood E Mendivil J Lambrelli D. Direct costs of unintended pregnancy in Spain, in European journal of contraception and reproductive. Health Care. (2015) 20:308–18. doi: 10.3109/13625187.2015.1028617, PMID: 25843298

[10] Prasad Sapkota V Dhakal L Adhikari SR. Economic burden of unintended pregnancies from societal perspective: a case of Nepal. Combined Issue Econ J Dev Issues. (2015) 2015:83–99.

[11] Skanes-DeVold H Maxwell RA Lindheim SR. Unintended pregnancy and contraceptive use: perceptions & prevention. J Contr Stu. (2015) 1:1.

[12] Damson EC, Modern contraception utilization among adolescent girls. (2020).

[13] Olson DJ Piller A. Ethiopia: an emerging family planning success story. Stu Family Plann. (2013) 44:445–59. doi: 10.1111/j.1728-4465.2013.00369.x 24323662

[14] Stanciole A Maurizio F. Estimating the health impacts and economic returns of increased family planning provision in Khyber-Pakhtunkhwa: A cost-benefit analysis. (2019).

[15] Stanciole A Maurizio F. Estimating the health impacts and economic returns of increased family planning provision in Balochistan: A cost-benefit analysis. (2019).

[16] Stanciole A Maurizio F. Estimating the health impacts and economic returns of increased family planning provision in Punjab: A cost-benefit analysis. (2019).

[17] Stanciole A Maurizio F. Estimating the health impacts and economic returns of increased family planning provision in Sindh: A cost-benefit analysis. (2019).

[18] International, M.S. Impact 2 an innovative tool for estimating the impact of reproductive health programmes. (2015).

[19] Weinberger MB Fry K Boler T Hopkins K. Estimating the contribution of a service delivery organisation to the national modern contraceptive prevalence rate: Marie stopes International’s impact 2 model. BMC Public Health. (2013) 13:1–17. 23902699 10.1186/1471-2458-13-S2-S5 PMC3684538

[20] Bongaarts J. A framework for analyzing the proximate determinants of fertility. Popul Dev Rev. (1978) 4:105–32. doi: 10.2307/1972149

[21] Houdroge F Yunus H Delport D Stearns E Palmer A Naim A. Cost optimisation analysis of the expanded programme for immunisation: balancing equity and coverage in Pakistan. BMJ Glob Health. (2022) 7:1–11. 10.1136/bmjgh-2022-009000 PMC9558787 36220307

[22] Hutton G Haller L. Evaluation of the costs and benefits of water and sanitation improvements at the global level. WHO. (2004). 10.2166/wh.2007.008 17878561

[23] Weissman E Mbonye AK Kayaga E Kihuguru SM Lissner C. Uganda safe motherhood programme costing study. Geneva: World Health Organization (1999).

[24] Korir J Kioko U. Family Planning Spending Assessment in Kenya. Nairobi: Centre for Economic and Social Research (2020).

[25] Idara-e-Taleem-o-Agahi. Annual status of education:ASER -PAKISTAN 2023. Lahore: Idara-e-Taleem-o-Agahi (2023).

[26] Science, P.A.f.M.a. The missing third of Pakistan: A tehsil-wise analysis of out of school children. Islamabad: Science, P.A.f.M.a (2024).

[27] Zuhdi A Firman F Ahmad R. The importance of education for humans. Indonesian J School Counseling. (2021) 6:22. doi: 10.23916/08742011

[28] Mardomi R Mardomi S. Early childhood education effect on sustainable development and developmental education. ECRJ. (2023) 6:64–72. doi: 10.23917/ecrj.v6i2.19482

[29] Nayab D. Demographic dividend or demographic threat in Pakistan. Pak Dev Rev. (2008) 47:1–26. doi: 10.30541/v47i1pp.1-26

[30] Khan A Khan AA. Can Pakistan reap its demographic dividend. Research and Development solutions policy brief series, vol. 3. Islamabad: Research and Development Solutions (2012).

[31] Census of Pakistan. Pakistan bureau of statistics. Islamabad: Census of Pakistan (2023).

[32] Monea E Thomas A. Unintended pregnancy and taxpayer spending. Perspect Sex Reprod Health. (2011) 43:88–93. 21651707 10.1363/4308811

[33] Wolfson LJ Gasse F Lee-Martin SP Lydon P Magan A Tibouti A. Estimating the costs of achieving the WHO-UNICEF global immunization vision and strategy, 2006-2015. Bull World Health Organ. (2008) 86:27–39. doi: 10.2471/BLT.07.045096, PMID: 18235887 PMC2647343

[34] Adnan M Xiao B Bibi S Xiao P Zhao P Wang H. Known and unknown environmental impacts related to climate changes in Pakistan: an under-recognized risk to local communities. Sustain For. (2024) 16:6108

[35] Black E Maidment RI Rees E Nderitu E. A new drought model for disaster risk management in the Punjab, Sindh and Baluchistan provinces of Pakistan. Front Clim. (2024) 6:1332233. doi: 10.3389/fclim.2024.1332233

[36] Mujtaba G Shah MUH Hai A Daud M Hayat M. A holistic approach to embracing the united Nation’s sustainable development goal (SDG-6) towards water security in Pakistan. J Water Proc Eng. (2024) 57:104691. doi: 10.1016/j.jwpe.2023.104691

[37] Frost JJ Sonfield A Zolna MR Finer LB. Return on investment: a fuller assessment of the benefits and cost savings of the US publicly funded family planning program. Milkbank Q.uarterly. (2014) 92:696–749. doi: 10.1111/1468-0009.12080, PMID: 25314928 PMC4266172

[38] Rana MJ Goli S. The road from ICPD to SDGs: health returns of reducing the unmet need for family planning in India. Midwifery. (2021) 103:103107. doi: 10.1016/j.midw.2021.103107 34358778

[39] Bellizzi S Pichierri G Menchini L Barry J Sotgiu G Bassat Q. The impact of underuse of modern methods of contraception among adolescents with unintended pregnancies in 12 low-and middle-income countries. J Global Health. (2019) 9:1–9. doi: 10.7189/jogh.09.020429, PMID: 31673342 PMC6815657

[40] Starbird E Norton M. Investing in family planning: key to achieving the sustainable development goals. PubMed Central. (2016) 22:191–210. doi: 10.9745/GHSP-D-15-00374 PMC4982245 27353614

[41] Khan AA Shujaat K. Revisiting the costs and utilization of family planning services in the public sector in Pakistan. J Pak Med Assoc. (2021) 71:S33–7. PMID: 34793426

[42] Khan AA Ahmad T Najam A Khan A. Community-driven family planning in urban slums: results from Rawalpindi, Pakistan. Biomed Res Int. (2023) 2023:7780. doi: 10.1155/2023/2587780, PMID: 36794253 PMC9925254

[43] Cleland J Conde-Agudelo A Peterson H Ross J Tsui A. Contraception and health. Lancet. (2012) 380:149–56. doi: 10.1016/S0140-6736(12)60609-6 22784533

[44] Erfani A. Family planning and women’s educational advancement in Tehran, Iran. Can Stud Popul. (2015) 42:35–52.

